# In Vitro and Ex Vivo Evaluation of Tablets Containing Piroxicam-Cyclodextrin Complexes for Buccal Delivery

**DOI:** 10.3390/pharmaceutics11080398

**Published:** 2019-08-08

**Authors:** Eleni Kontogiannidou, Martina Ferrari, Asteria-Danai Deligianni, Nikolaos Bouropoulos, Dimitrios A. Andreadis, Milena Sorrenti, Laura Catenacci, Kazem Nazari, Muhammad Sohail Arshad, Ming-Wei Chang, Zeeshan Ahmad, Dimitrios G. Fatouros

**Affiliations:** 1 Laboratory of Pharmaceutical Technology, Department of Pharmacy, Aristotle University of Thessaloniki, GR-54124 Thessaloniki, Greece; 2 Laboratory of Physical Pharmacy, Department of Drug Science, University of Pavia, 27100 Pavia, Italy; 3 Department of Materials Science, University of Patras, 26504 Patras, Greece; 4 Foundation for Research and Technology Hellas, Institute of Chemical Engineering and High Temperature Chemical Processes, 26504 Patras, Greece; 5 Department of Oral Medicine/Pathology, School of Dentistry, Aristotle University of Thessaloniki, GR-54124 Thessaloniki, Greece; 6 The Leicester School of Pharmacy, De Montfort University, The Gateway, Leicester LE1 9BH, UK; 7 College of Biomedical Engineering and Instrument Science, Zhejiang University, Hangzhou 310027, China; 8 Zhejiang Provincial Key Laboratory of Cardio-Cerebral Vascular Detection Technology and Medicinal Effectiveness Appraisal, Zhejiang University, Hangzhou 310027, China; 9 Nanotechnology and Integrated Bioengineering Centre, University of Ulster, Jordanstown Campus, Newtownabbey, Northern Ireland BT37 0QB, UK

**Keywords:** cyclodextrins, in vitro studies, ex vivo buccal permeation

## Abstract

In the current study, the development of mucoadhesive tablets for buccal delivery of a non-steroidal anti-inflammatory drug was investigated. Binary complexes with piroxicam and cyclodextrins (β-cyclodextrin (β-CD), methylated-β-cyclodextrin (Me-β-CD), and hydroxypropyl-β-cyclodextrin (HP-β-CD)) were prepared by the co-evaporation method. All formulations were characterized by means of differential scanning calorimetry, infrared spectroscopy and powder X-ray diffractometry. Mucoadhesive tablets of binary systems were formulated by direct compression using chitosan as mucoadhesive polymer. The in vitro release profiles of tablets were conducted in simulated saliva and, the drug permeation studies, across porcine buccal mucosa. The results suggest that the rank order effect of cyclodextrins for the drug release was Me-β-CD > HP-β-CD > β-CD, whereas the ex vivo studies showed that the tablets containing chitosan significantly increased the transport of the drug compared to their free complexes. Finally, histological assessment revealed loss of the superficial cell layers, which might be attributed to the presence of cyclodextrins.

## 1. Introduction

Buccal mucosa is an alternative site for the delivery of drugs into the systemic circulation [1]. The drug administered through the buccal mucosa enters to the systemic circulation directly via the jugular vein [2], minimizing the first-pass hepatic metabolism, and avoiding the adverse gastro-intestinal tract [3]. Buccal delivery of active pharmaceutical ingredients (APIs) is an alternative, non-invasive route of administration that provides beneficial health effects. Buccal mucosal has a rich vascularization and a high permeability for many APIs in addition to the fact that first-pass effect is avoided [4]. Several drug delivery systems intended for buccal administration have been developed. Most common are films, patches and mucoadhesive tablets. However, oral mucosa permeability of the drugs is too low to allow plasma concentration to reach therapeutic levels [5]. There are two major routes involved: Transcellular and paracellular route. For buccal mucosa, it is thought that the majority of drugs are transported through the paracellular route by passive diffusion [6].

Cyclodextrins (CDs) are a group of cyclic oligosaccharides composed by α-d-glucopyranose units linked together with α-1,4-glycosidic bonds. They possess a relatively hydrophobic central cavity, which provides a microenvironment for appropriately sized nonpolar molecules, and a hydrophilic outer surface, which makes them water-soluble. CDs have attracted interest in the pharmaceutical field because of their ability to modify physical, chemical and biological properties of many hydrophobic drug molecules through the formation of inclusion complexes [7,8]. Through inclusion complex formation they can increase, substantially, the solubility and ultimately the bioavailability of the APIs [9,10]. CDs are enrolled as a means to increase the solubility of drugs with poor solubility because of the improved characteristics of the complexes, such as aqueous solubility, chemical stability, and bioavailability [11]. They act as a dissolution enhancer for the insoluble drug. It is known that they can be used as permeation enhancers (by skin, mucosa, and cornea) at the membrane partition without disrupting the lipid layers of the barrier. It was found that the addition of polymers can further enhance the drug permeability from aqueous cyclodextrin solutions [12].

Piroxicam (PX) is a non-steroidal anti-inflammatory drug (NSAID) belonging to the new oxicam family and it is a non-selective cyclooxygenase (COX) inhibitor. It is used to reduce the pain, inflammation, and stiffness caused by rheumatoid arthritis and osteoarthritis. It also possesses analgesic and antipyretic properties [13]. PX has a low water solubility, a high membrane permeability, and it is classified as Class II drug in Biopharmaceutics Classification System. Different techniques have been studied to enhance the dissolution rate of piroxicam and improve its oral bioavailability. These strategies mainly include solid dispersion with some carriers and polymers (like cyclodextrins), particle size reduction via micronization, and the conversion of the crystalline molecule to its amorphous state [14].

The main aim of this work was to evaluate the influence of cyclodextrins (β-CD, Me-β-CD, HP-β-CD) on the drug dissolution [15,16] and its permeability through buccal porcine mucosa. Raw materials and cyclodextrin complexes were characterized by differential scanning calorimetry (DSC), infrared spectroscopy (ATR-IR), and X-ray diffractometry (XRD) [17,18,19]. Buccal mucoadhesive tablets were prepared by direct compression using chitosan as bioadhesive polymer [20]. Chitosan has been used as an excipient for direct tableting to enhance the dissolution properties of insoluble drugs and to prepare sustained release formulation [21]. The drug release behavior was evaluated in vitro and ex vivo. The porcine buccal epithelium is the most frequently utilized model due to its relative similarity to human tissue. Some similarities are the thickness, the nature of keratinization, and the lipid composition. The low cost associated with tissue acquisition is also important [5]. Finally, the buccal mucosa examination was performed to assess the histological changes resulting from the presence of the cyclodextrins and chitosan. 

## 2. Materials and Methods

### 2.1. Materials 

Piroxicam (PX) and low molecular weight chitosan (LMW, 150 kDa, DD 95–98%) were obtained from Sigma Aldrich (Munich, Germany). β-cyclodextrin (β-CD), methylated-β-cyclodextrin (number of methyl groups per molecule (DS) = 0.57) and hydroxypropyl-β-cyclodextrin (average number of hydroxypropyl groups per anhydroglucose unit (MS) = 0.85) KLEPTOSE^®^ were purchased from Roquette (Lestrem, France), with MW 1135, 1191 and 1376 g/mol, respectively. All chemicals and solvents were of analytical grade.

Simulating saliva buffer (pH 6.8) was prepared using sodium chloride (0.8 g), sodium phosphate dibasic (2.38 g), and potassium phosphate monobasic (0.19 g) in 1 L of distilled water. Phosphate buffer saline (PBS) pH 7.4 was prepared by dissolving sodium chloride (8.0 g), potassium chloride (0.20 g), sodium phosphate dibasic (1.44 g), and potassium phosphate monobasic (0.24 g) in 1 L of distilled water. All components of buffer solutions were purchased from Merck (Darmstadt, Germany).

### 2.2. Methods

#### 2.2.1. Preparation of Solid Complexes

Complexes were prepared in 1:1 piroxicam:cyclodextrins (PX:CDs) molar ratio, based on previous results of solubility studies [14,22]. Physical mixtures (piroxicam and cyclodextrins) were passed through 0.5 mm meshed and were homogeneously blended in Turbula V mixer for 20 min. In addition, piroxicam and an equimolar amount of CDs were dissolved in distilled water by addition of a 25% *v*/*v* ammonium hydroxide solution, for preparing co-evaporation products (RV). The solution was stirred for 24 h at ambient temperature, and subsequently was subjected to evaporation using a rotary evaporator. 

#### 2.2.2. Tablet Preparation

The tablets were prepared by direct compression of RV drug-CDs complexes with excipients. Chitosan (LMW, 7 mg) was used as mucoadhesive, lactose monohydrate (60 mg) was used as diluent, and magnesium stearate (5 mg) was used as lubricant. The powder was gently mixed in a mortar and then directly compressed using a 10 mm single punch eccentric tablet press machine. The compression force was 10 kN. Each tablet (100 mg) contained 5 mg of piroxicam, in complex form.

#### 2.2.3. Drug Loading and Water Uptake Studies

The obtained drug-loaded complexes (1 mg) were dispersed in 5 mL of PBS pH 7.4 (with drops of NH_4_OH solution) in sealed glass vials and kept under stirring (200 rpm) for 30 min at room temperature. The resulting solutions were centrifuged at 4500 rpm for 15 min, and quantification of piroxicam by means of UV-analysis at 357 nm (UV–Vis spectrophotometer, Shimadzu, UVmini-1240, Kyoto, Japan). The drug content was calculated according to the following equation:

Drug content (% W/W) = 100 × (W_drug_/W_drug loaded complexes_) (1)

Furthermore, encapsulation efficiency was defined as the ratio of actual and original amount of piroxicam encapsulated in CDs complexes:

Encapsulation Efficiency (% EE) = 100 × (Actual amount of drug in CDs complexes/Theor. amount of drug in CDs complexes)(2)

Water uptake of the tablets was determined gravimetrically in a phosphate buffer (pH 6.8). The pre-weighed tablets were immersed in Petri dishes containing 400 μL of buffer. At predetermined times, the tablets were removed from the media, wiped carefully to remove excess water, and weighed. Water uptake was calculated according to the equation:

Water uptake (%) = 100 × ((W_after the immersion_ − W_before the immersion_)/W_before the immersion_) (3)

The swelling rates were determined as the slope of plots Water Uptake (%) versus time.

#### 2.2.4. Physicochemical Characterization

Differential scanning calorimetry (DSC) thermograms of drug, CDs, and their solid binary systems were performed using a DSC 204 F1 Phoenix (Netzsch, Selb, Germany) instrument. All samples (5 mg) were placed in sealed aluminum pans. The thermograms were recorded in a temperature range from 30 to 250 °C with a heating rate of 10 °C/min under a nitrogen purge of 70 mL/min. 

X-ray diffraction studies (XRD) were performed to evaluate the crystallinity of the samples prepared. XRD analysis was performed on a Bruker D8-Advance diffractometer equipped with a LynxEye type detector (Bruker AXS, Karlsruhe, Germany) and using Cu Kα radiation (λ = 1.5421 Å, 40 kV, 40 mA). Spectra was collected over the 2θ range from 5° to 50° at a scanning rate of 0.35 s/step and a step size of 0.02°.

The characterization of the samples was supported by Fourier transform infrared spectroscopy (ATFT-IR). Infrared spectra were employed to investigate the interaction of CDs with PX. Analysis of the samples was carried out using a spectrophotometer ATR-FTIR (Prestige-21, Shimadzu, Kyoto, Japan) in the range between 650 and 4000 cm^−1^ with resolution of 4 cm^−1^ after 64 scans.

#### 2.2.5. In Vitro Release Studies

##### Dissolution Studies

Dissolution tests of tablets were carried out with apparatus using the paddle method (USP Type II). The tablets were kept in metal grids, fixed with metal clamps, and immersed in the bottom of the vessel. Simulating saliva buffer solution (pH 6.8) was used as the dissolution medium, under sink conditions at 37 °C. The paddle speed was 50 rpm and the dissolution volume was 500 mL. At predetermined time intervals (5, 10, 15, 30, 45, 60, 90, and 120 min), aliquots of 1 mL were withdrawn and replaced with fresh buffer to keep the volume constant. The samples were filtered by a 0.45 μm PTFE syringe prior to analysis by UV-Vis at 357 nm. A good linearity (*r*^2^ = 0.999) was established in the range of 1–20 μg/mL. Drug release kinetics was evaluated in SigmaPlot v.12.5 (Systat Software, Inc., Chicago, IL, USA) using Korsmeyer–Peppas, Higuchi, and first-order kinetic models.

##### Permeation Studies

Freshly mucosal specimens were obtained from slaughtered pigs on the day of the experiment. The tissues were transported in ice cold PBS (pH 7.4) to the laboratory. Buccal mucosa was taken from the region posterior to the angle of the mouth. The epithelium was carefully separated from the underlying tissue using forceps and scissors within 1 h after excision. The thickness of the slices ranged from 600 to 850 μm. The buccal tissues were placed in vertical Franz type diffusion cells with diffusion area of 4.9 cm^2^, acceptor volume of 15 mL of degassed PBS (pH 7.4), and the donor compartment was filled with the tablets or the free complexes with the same amount of drug, in 1 mL of simulating saliva buffer solution. The temperature was set at 37 °C under stirring at 100 rpm. Samples of 1 mL were withdrawn from the receptor phase, over a period of 5 h (at 1, 2, 3, 4, and 5 h, respectively) and replaced with fresh PBS. They were further analyzed by UV-Vis as described above. All experiments were carried under occlusive conditions and results are reported as means of five different repetitions. Blank experiments containing only the medium were performed and any interference was subtracted.

Steady state flux (J_ss_) was calculated from the slope of the linear section of the line obtained after plotting the amount of the drug permeating buccal mucosa per unit area (μg/cm^2^) against time (h). The apparent permeability coefficient (P_app_) was calculated using Equation (4), where C_d_ is the initial concentration of the drug in the donor compartment and J_ss_ is the steady state flux.

P_app_ = J_ss_/C_d_ (cm/h) (4)

#### 2.2.6. Histological Evaluation

After ex vivo studies, each tissue was fixed in buffered 10% formalin and embedded in paraffin wax. Four-micrometer-thick sections of the tissue were stained with hematoxylin-eosin and were placed on microscope slides. The tissue sections were analyzed with an Olympus CX31 optical microscope and images were processed with OLYMPUS analysis getIT software (Version 5-1, Shinjuku-ku, Tokyo, Japan).

#### 2.2.7. Statistical Analysis

All values of the studies are presented as means. ± S.D. Student’s *t*-test was used for statistical analysis of data and statistical significance is indicated by *p* < 0.05 values.

## 3. Results and Discussion

### 3.1. Drug Content Quantification and Characterization Studies

Drug content quantification of CDs complexes is illustrated in Table 1. No distinctive differences were observed among binary systems, and good encapsulation efficiency was achieved (85% for β-CD, 96% for Me-β-CD and 94% for HP-β-CD).

**Figure 1:**
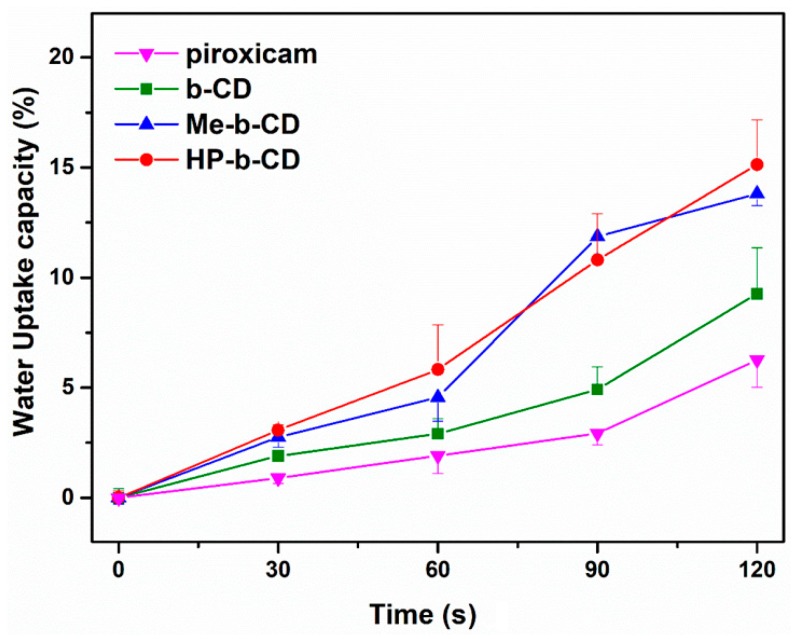
The water uptake capacity of tablets (*n* = 3).

The water uptake results are illustrated in Figure 1. The capability of tablets to absorb water molecules was feasible to measure up to 2 min, as immersion in the medium for a longer time period resulted in erosion of the formulation. All the formulations showed an increase in weight due to water uptake. According to previous reports [14], complex formations could increase molecular mobility and release more sites for hydrogen bonding with water. 

**Figure 2:**
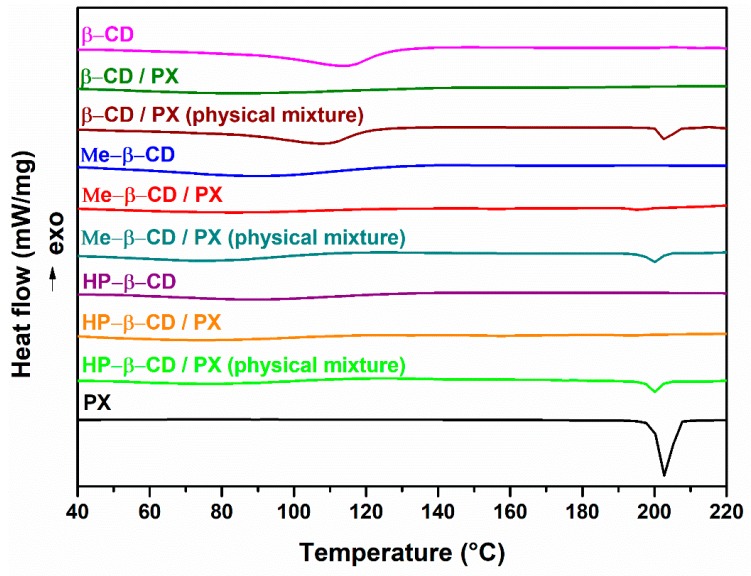
DSC thermograms of pure compounds and binary systems.

DSC thermograms of pure components and the various binary systems are shown in Figure 2. The curve of PX showed an endothermic peak at 202 °C, corresponding to the melting of a cubic crystal polymorph form (polymorph I) of the drug [15,22]. This endothermic effect was also evident in the thermogram of physical mixtures, indicating no interaction between drug and carrier in these systems. Thermal behavior of CDs showed dehydratation phenomena, seen as a wide endothermic peak in a different range of temperature for each cyclodextrins (β-CD 100–130 °C, Me-β-CD 70–110 °C, HP-β-CD 60–120 °C). β-CD binary complex showed, in the temperature 70–110 °C, the solvent exit seen as a wide endothermic peak shifted to lower temperature in RV products. The dehydration effect in the RV product with β-CD is shifted to lower temperature 70–100 °C, and appeared to be of lower intensity because of the dehydration due to the RV treatment. The disappearance of PX endothermic melting peak in the thermogram of the RV product can be attributed to the inclusion of drug in the cyclodextrin cavity [23]. DSC curve of the Me-β-CD complex showed, for each curve, the solvent loss in a range temperature between 70–110 °C. The shifting of the melting peak of the PX to a lower temperature, accompanied with enthalpy values reduction, confirmed the interaction of PX with CD and/or a partial amorphization of the drug [15]. Thermograms of HP-β-CD showed the same thermal profile of dehydratation (60–100 °C) seen in the previous system and the disappearance of the melting peak of the pure drug in RV products. Furthermore, these findings for RV products could show an in situ inclusion complex formation, according to other studies involving drugs inclusion complexes with CDs [24,25].

**Figure 3:**
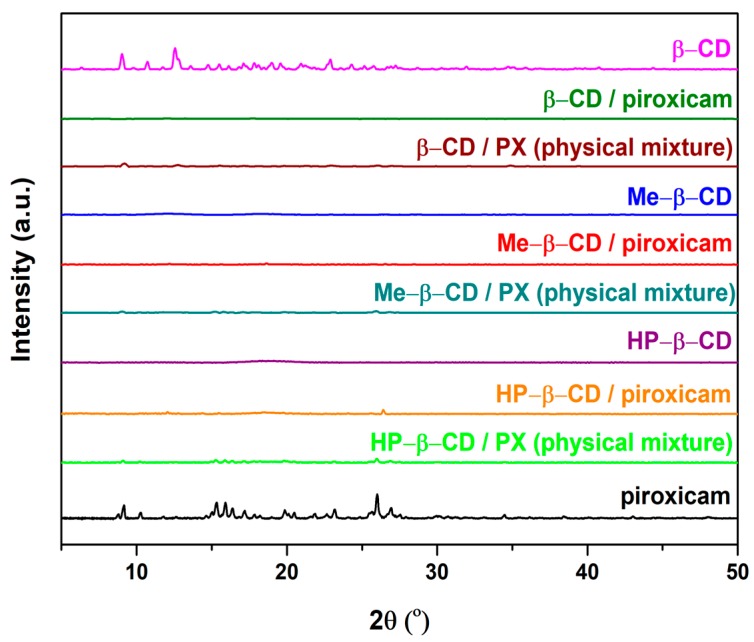
XRD patterns of pure compounds and binary systems.

The X-ray diffractometry studies (Figure 3) confirm the crystalline nature of PX and β-CD, as well as the amorphous state of Me-β-CD and HP-β-CD. PX have a number of sharp peaks at 9.1°, 10.3°, 15.2°, 15.9°, 16.4°, 17.2°, 19.8°, 23.1°, 26.4°, and 27.1° [26]. Some of these peaks are already present in the diffractograms of physical mixtures, but appear of lower intensity compared to those of the pure drug. The diffractograms of the RV products showed no drug’s crystal signals, demonstrating the amorphous state of products probably due to their protection of PX in the cavity of CDs cyclodextrins. The results obtained in the current study are in agreement with previous studies reporting on the complexation of Hp-β-CD [14], Me-β-CD, and β-CD with PX [24].

**Figure 4:**
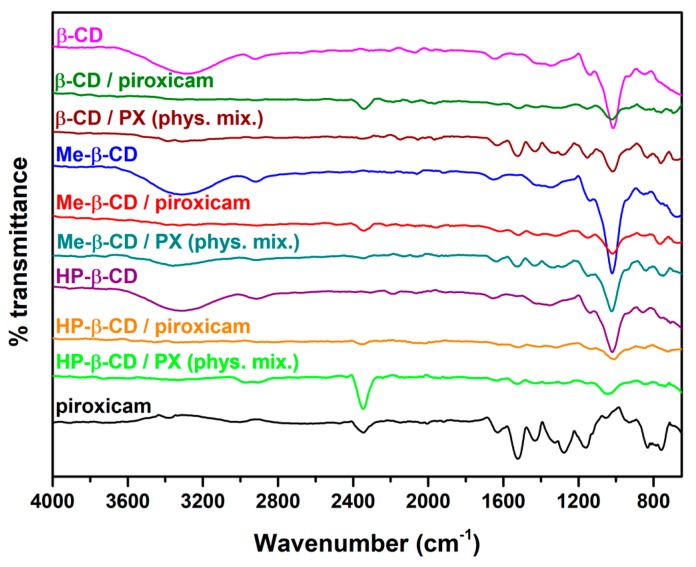
FTIR spectra of pure components and binary systems.

FT-IR spectra are illustrated in Figure 4. PX exhibits characteristic bands at 3330 cm^−1^ (-NH stretching), 2970 cm^−1^ (-OH stretching), and 1630 cm^−1^ (C=O group of the cubic form) [15]. The spectra of CDs present a profile without distinctly high peaks in the range of 3000–3700 cm^−1^ (–OH groups) and in the range of 2800–3000 cm^−1^ (C-H groups) [14,26,27]. Physical mixtures shows the presence of both components (PX and CD). The FI-IR spectra of native Hp-β-CD, Me-β-CD, and β-CD are in broad agreement with previous reported data [14,24], whereas characteristic peaks attributed to the presence of PXM are clearly visible in the dominant physical mixtures. In the case of the inclusion complexes, broadened peaks are the dominating features in the spectra in accordance with previous studies [14,24].

### 3.2. In Vitro Release

**Figure 5:**
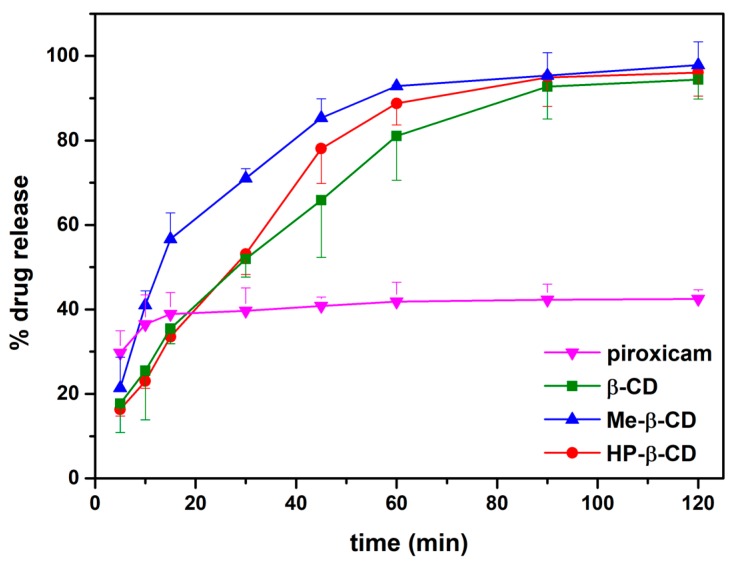
In vitro release studies of pure piroxicam and binary systems in tablet form (*n* = 3).

The release profiles of PX from tablets are shown in Figure 5. A steep release of the pure drug was noticed. After 15 min about 40% of the initial amount of the drug was released attaining a plateau for the rest of the time period of the study. The release behavior of PX from tablets increases over time, resulting in prolonged release [14]. A total amount of about 96% was dissolved within 2 h (94% for β-CD, 96% for HP-β-CD, and 98% for Me-β-CD). The enhanced release rate of PX from tablets might be due to the decrease in crystallinity of inclusion complexes. The results demonstrated that the presence of cyclodextrins had no effect on the release rate of the active (*t*-test, *p* > 0.05). 

The kinetic models were applied to the release data and the parameters are presented in Table 2. In the first-order model, all developed formulations show high linearity with *R*^2^ (0.98–0.99), compared to Korsmeyer-Peppas model (*R*^2^ = 0.91–0.97). Moreover, the release rate (k) was different to the Korsmeyer-Peppas model for each formulation, in contrast with first-order, where k values were similar.

### 3.3. Drug Permeation

**Figure 6:**
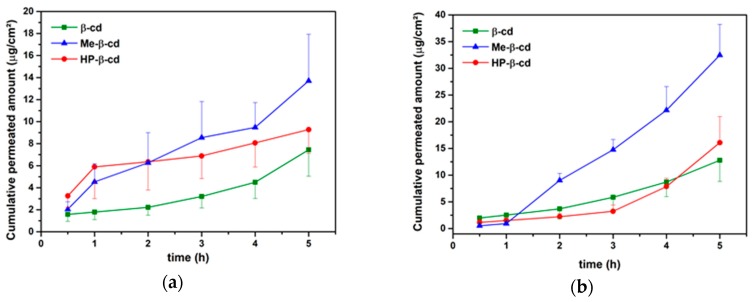
Cumulative transport of piroxicam across buccal mucosa (**a**) in free complexes and (**b**) in tablet form.

Table 3 shows the permeation parameters of the study and the permeation profiles are illustrated in Figure 6a,b. The rank order effect of the CDs for the transport of PX across buccal epithelium was Me-β-CD > HP-β-CD > β-CD [28]. However, only the values between β-CD and Me-β-CD were statistical different (*t*-test, *p* < 0.05). Lipophilic CDs, such as Me-β-CD, have the ability to remove lipids from cell membranes forming complexes. They also can act as permeation for drugs through the buccal mucosa [8]. However, β-CD may retain the drug onto the surface of epithelium. This rank order was also maintained for free complexes and tablets, but the value of the permeability coefficient in tablet form was approximately 3-fold higher than that measured for every CD *t*-test at *p* < 0.05. This result is due to the presence of chitosan which might have a synergistic effect with cylcodextrins resulting in an enhanced permeation of PXM across porcine buccal epithelium compared to the free complexes, for all types of CDs tested. 

### 3.4. Histological Studies

**Figure 7:**
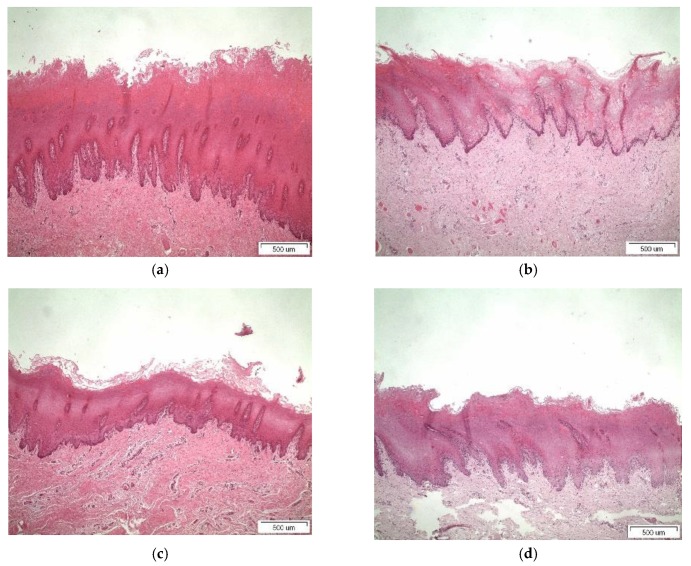
Light micrographs of (**a**) porcine buccal mucosa (untreated); (**b**) free complex with β-CD); (**c**) free complex with Me-β-CD; and (**d**) free complex with HP-β-CD. Bar represents 500 μm.

**Figure 8:**
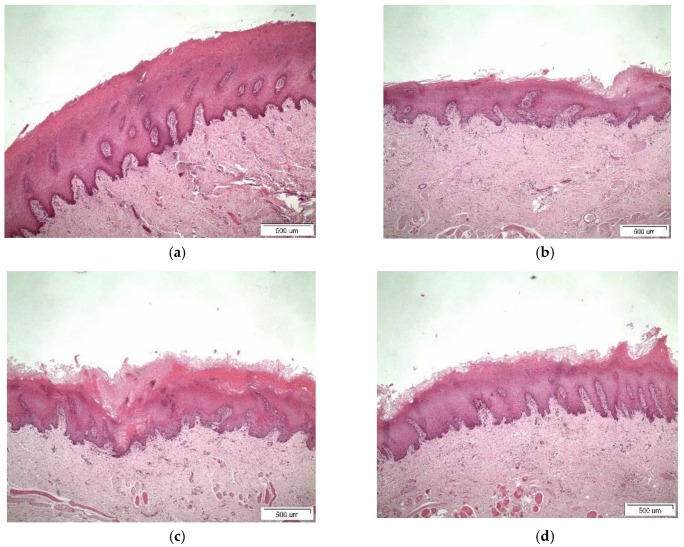
Light micrographs of tablet with (**a**) chitosan as control (without complexes); (**b**) β-CD; (**c**) Me-β-CD; and (**d**) HP-β-CD. Bar represents 500 μm.

Light micrographs of porcine buccal tissue after 5 h treatment with binary systems and tablets are shown in Figure 7 and Figure 8, respectively. For β-CD in Figure 7b and Figure 8b, mild desquamation of superficial epithelium was observed. Detachment of the epithelium from underlying stroma and mild disorganization at basal membrane are illustrated in the micrographs of Figure 7c and Figure 8c for Me-β-CD, with only rare acantholysis and inter-epithelial gaps in tablet form. In Figure 7d and Figure 8d, desquamation of superficial keratinocytes and mild disorganization of collagen beneath basal membrane could be identified for HP-β-CD [3,8,29]. The loss of the superficial cell layers might imply interactions between the CDs and the epithelial lipids given that CDs can induce lipid solubilization [30]. The histological findings show swelling when permeation enhancers are present and the effect of CDs further corroborated the ex vivo results. The results obtained from the histological evaluation further corroborate the in vitro permeation, where in the case of free complexes, the highest transport of PXM were recorded for Μe-β-CD, accompanied by extended detachment of the superficial layers compared to its congeners β-CD and Hp-β-CD. This might be attributed to the affinity of lipophilic cyclodextrins to biological barriers, altering the membrane permeability by opening the tight junctions. In a similar manner the same pattern was observed for the tablets as well.

## 4. Conclusions

The results from the current study demonstrate that the complexation of piroxicam with cyclodextrins could be used to provide controlled drug release in vitro and increase the permeation of the drug across buccal mucosa. The combination of chitosan and Me-β-CD can substantially increase the transport of PXM across porcine buccal epithelium, whereas the changes observed in epithelium were mainly restricted to the upper non-keratinized epithelial cells.

## Figures and Tables

**Table 1:** Drug content and encapsulation efficiency of solid complexes.

Samples	Drug Content (%) ± SD	Encapsulation Efficiency (%)
β-CD	19.34 ± 0.93	85.57%
Me-β-CD	18.49 ± 0.92	96.51%
HP-β-CD	20.58 ± 0.95	94.66%

^1^ Values are reported as mean ± standard deviation (*n* = 3).

**Table 2:** Curve fitting parameters.

Samples (Tablets)	Korsmeyer-Peppas Model			First Order Model	
	*k*	*n*	*R* ^2^	*k*	*R* ^2^
β-CD	9.64 ± 0.05	0.49 ± 0.04	0.97	0.03 ± 0.002	0.99
Me-β-CD	20.17 ± 0.03	0.34 ± 0.02	0.91	0.05 ± 0.007	0.98
HP-β-CD	9.88 ± 0.07	0.49 ± 0.08	0.93	0.03 ± 0.003	0.99

**Table 3:** Calculated steady state flux values (J_ss_) and permeability coefficients (P_app_) of free complexes and tablet form.

Samples	J_ss_ (μg/cm^2^ h)	P_app_ (× 10^−3^) (cm/h)
β-CD	1.165 ± 0.44	0.21 ± 0.08
Me-β-CD	2.405 ± 0.87	0.42 ± 0.12
HP-β-CD	1.850 ± 0.43	0.30 ± 0.07
β-CD (tablet)	3.019 ± 0.68	0.51 ± 0.14
Me-β-CD (tablet)	7.778 ± 1.69	1.26 ± 0.35
HP-β-CD (tablet)	5.438 ± 1.04	0.94 ± 0.34

^1^ Values are reported as mean ± standard deviation (n = 5).
